# Evaluation of Permulgin 3274 as a Material for the Conservation of Beeswax Seals

**DOI:** 10.3390/ma15051909

**Published:** 2022-03-04

**Authors:** Lenka Bílková, Benjamin Bartl, Štěpán Urbánek, Martin Zapletal, Libuše Holakovská, Michal Ďurovič, Zdeněk Hrdlička, Jakub Havlín

**Affiliations:** 1 National Archives, Archivní 2257/4, 149 00 Prague 4, Czech Republic; lenka.bilkova@nacr.cz (L.B.); stepan.urbanek@nacr.cz (Š.U.); libuse.holakovska@nacr.cz (L.H.); 2 Department of Organic Technology, Faculty of Chemical Technology, University of Chemistry and Technology, Technická 5, 166 28 Prague 6, Czech Republic; martin.zapletal@vscht.cz; 3 Department of Chemical Technology of Monument Conservation, Faculty of Chemical Technology, University of Chemistry and Technology, Technická 5, 166 28 Prague 6, Czech Republic; michal.durovic@vscht.cz; 4 Department of Polymers, Faculty of Chemical Technology, University of Chemistry and Technology, Technická 5, 166 28 Prague 6, Czech Republic; zdenek.hrdlicka@vscht.cz; 5 Laboratory of Thermal-Gravimetric Analysis, Faculty of Chemical Technology, University of Chemistry and Technology, Technická 5, 166 28 Prague 6, Czech Republic; jakub.havlin@vscht.cz

**Keywords:** Permulgin 3274, ceresin, beeswax, seal, conservation, ageing

## Abstract

When treating historical beeswax seals, it seems a natural choice to use materials as similar to the original as possible. The properties of analogous recent materials, however, differ from those of the aged ones, not to mention the fact that the exact composition of the particular sealing wax is usually uncertain. In order to obtain the material of desired properties, recent beeswax is often combined with various additives, including petroleum waxes, or even replaced by mixtures based solely on these products. Within this study, the relevant properties of Permulgin 3274, a ceresin-type wax, were compared with the characteristics of recent and historical beeswaxes. The aim was to evaluate its advantages and limitations, in terms of its possible use for the conservation of beeswax seals. The properties studied were comprised of the chemical composition, thermal properties, mechanical properties, possibilities of colour adjustment and ageing properties. Permulgin 3274′s workability was evaluated by conservators from the National Archives in Prague. The results indicate that, from the technological point of view, Permulgin 3274 could be considered a welcome alternative to the use of traditional conservation mixtures.

## 1. Introduction

Historical seals are bearers of legal and cultural-historical values. An integral part of documents, they verify their validity, as a means of closing letters or other objects, they confirm the authenticity of their content, thus, protecting it from possible misuse. The imprinted images are also a valuable source of information within the field of heraldry, architecture, iconography or contemporary realities. Finally, their artistic and handicraft processing can reach such a level that it is possible to regard them as small pieces of plastic art.

**Figure 1:**
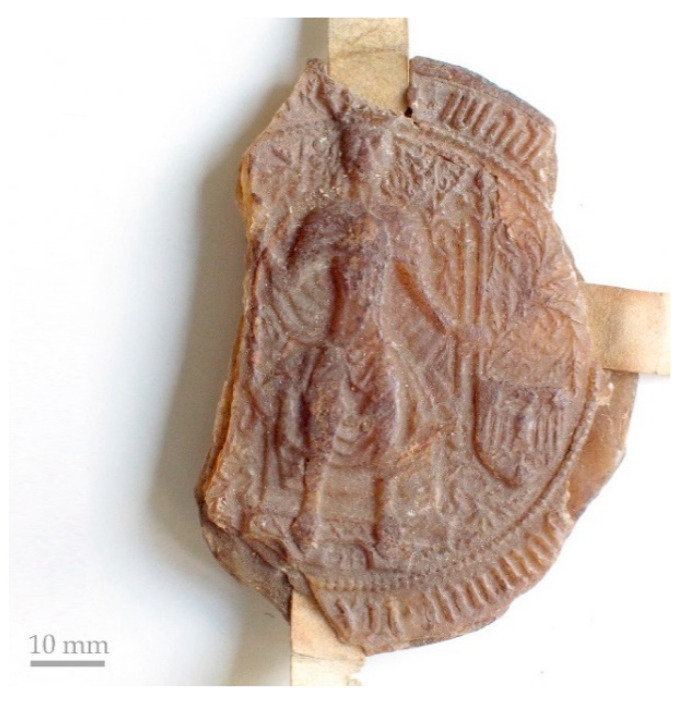
Damaged beeswax seal of the charter RM 722, dated 1495. Photograph courtesy of the Grand Priory of Bohemia of the Order of Malta.

Unfortunately, many of those unique objects are found in a poor state of preservation at present, often fragmented and incomplete (Figure 1). For conservators treating damaged beeswax-based seals, it might seem a natural choice to use material as similar to the original as possible. However, the exact composition of the particular sealing waxes is usually uncertain; moreover, the properties of analogous recent materials usually differ from those of the aged ones [1]. For example, recent beeswax is significantly softer than historical sealing wax and, as a consequence, is more prone to deformation, abrasion and soiling. Moreover, its natural colour is highly unstable and the appearance of its surface is often affected by the “wax bloom” phenomenon, the formation of the whitish efflorescence [2,3].

In order to obtain the material of the desired properties, recent beeswax is usually combined with various additives. The most common is the addition of natural resin, such as dammar resin or colophony. The amount added usually does not exceed 20% (*w/w*) [4,5,6,7,8,9,10,11,12,13]. For increasing the hardness of the mixture, the addition of up to 10% of carnauba wax is sometimes recommended [9,12]. When necessary, the workability of the material is improved by the addition of Venetian turpentine, drying vegetable oils or similar products [4,5,8,9,12]. Besides, conservation recipes often include additional components, e.g., stearin, spermaceti or pigments [4,6,9,12]. Beeswax is also combined with petroleum waxes, or even replaced by mixtures based solely on these products. The reason for their use is usually their assumed stability, favourable mechanical properties and distinguishability from the original material [5,14,15,16].

Petroleum (mineral) waxes represent a broad group of materials, comprising paraffins, microcrystalline waxes and ceresin-type waxes. All these consist mainly of linear, branched and cyclic saturated hydrocarbons, included in different proportions. While shorter linear chains predominate in paraffins, the high content of long-branched and cyclic chains is typical for microcrystalline waxes. This results in their microcrystalline structure, different mechanical properties, but also in a higher proportion of residual oil and other impurities. Common ceresin-type waxes are artificial mixtures of hydrocarbons, the properties of which resemble those of “genuine” ceresin, which in the past, was obtained by refining natural ozokerite [17,18].

The thermal and mechanical properties of different petroleum waxes vary considerably. Therefore, through careful selection or combination of these waxes, it is often possible to obtain a material with the desired properties. The paraffinic component is generally responsible for the increase in the crystallinity, hardness, brittleness and translucency of the mixture. The microcrystalline components usually contribute to the ductility and tack of the material. Ceresin-type waxes combine the advantages of both groups, to a certain extent. They can be characterized as brittle microcrystalline materials and were historically used as a cheaper substitute of beeswax in many applications since the 19th century [19].

Within this study, Permulgin 3274, a ceresin-type petroleum wax, was thoroughly characterized and its relevant properties were compared with those of recent and historical beeswax. This product was preselected on the basis of available data, regarding its composition, purity and congealing point. The aim was to evaluate its advantages and limitations in terms of its possible use for the conservation of beeswax seals.

## 2. Materials and Methods

The selection of Permulgin 3274 and the experiment‘s design was based on the following assumptions: Any material used for the conservation of historical seals should be primarily technologically compatible with the original sealing wax, reasonably stable and preferably distinguishable from the original. In addition, it should be processable by common conservation methods. Nowadays, a general trend to minimize conservation interventions can be recognized, and therefore the use of conservation wax mixtures is usually restricted to relatively thin layers, which essentially work as a kind of sealant. The reconstruction of the fragmented seal to its original shape and size is rather exceptional. It follows that the quality of the repair surface is of primary practical importance.

### 2.1. Samples

The commercially available ceresin-type wax Permulgin 3274 (E00136, lot: 18–53, Koster Keunen, Inc., Bladel, The Netherlands) was the main material of interest in this study. However, its refined, fully hydrogenated derivative was also used as a reference material. The hydrogenation was carried out at 100 °C and a hydrogen pressure of 10 MPa for 9 h. As a catalyst, 3% palladium on activated carbon was used. After the completion of the reaction, the catalyst was removed from the material by in-line filtration.

As the additional reference materials, recent food grade beeswax (Beekeeping Research Institute, Libčice nad Vltavou, Czech Republic) and its hydrogenated derivative were used. Hydrogenated beeswax was used as a model for historical beeswax, naturally aged for approximately 800 years [1]. The hydrogenation proceeded at 90 °C and a hydrogen pressure of 5 MPa for 60 min. The other details of the procedure remained the same. The reaction was terminated at the moment when no unsaturated compounds could be detected in beeswax by the GC-MS analysis.

### 2.2. Methods

#### 2.2.1. Chemical Composition

The chemical composition of the samples was analyzed by the GC-MS method. The samples were studied both in their original state and as trimethylsilylated derivatives. The aim of the modification was to facilitate the detection of possible traces of polar compounds in the samples. The derivatization was achieved using a BSTFA + 1% TMCS reagent and proceeded at 60 °C for 30 min. As internal standards, hexadecane and tridecanoic acid were added to the samples, in order to control the injection volume and the efficiency of the derivatization. All of the chemicals mentioned within the experimental section were obtained from Merck Life Science spol. s r.o., Prague, Czech Republic, unless otherwise specified. 

The separation was carried out using the GC-2010 chromatograph (Shimadzu Europa GmbH, Duisburg, Germany) equipped with the Supelco SLB-5ms capillary column (15 m length, 0.25 mm inner diameter, 0.25 μm film thickness, Merck Life Science spol. s r.o., Prague, Czech Republic). The injector was used in the split mode (10.0). At the beginning of each run, the temperature was held at 100 °C for 3 min, then gradually increased at a rate of 10 °C·min^−1^ to 350 °C, and subsequently held constant until the end of the run. Helium was used as a carrier gas. 

The mass spectra were obtained using the QP-2010 mass spectrometer (Shimadzu Europa GmbH, Duisburg, Germany), operating in an electron impact mode at 70 eV. The scanning frequency was 4 s^−1^. Both TIC and SIM modes were used in separate runs. The whole procedure generally followed the recommendation of Lluveras [20]. 

The individual groups of hydrocarbons were distinguished in SIM mode on the basis of the increased content of the following diagnostic fragments: *m*/*z* = 71, 85 for isoalkanes, *m*/*z* = 68, 69 for alkylcyclopentanes and *m*/*z* = 82, 83 for alkylcyclohexanes [21,22]. *n*-Alkanes were identified by comparing their retention times with those of standards (*n*-tritriacontane) and waxes of known hydrocarbon distribution (beeswax, Paraffin 62400, Kremer Pigmente GmbH & Co. KG, Aichstetten, Germany).

The total acid number is a measure of the quantity of acidic compounds present in a sample. It is expressed in milligrams of potassium hydroxide, which is required to neutralize all acidic constituents in 1 g of sample. Total acid number was determined following the standard method of ASTM D974. 

The measure of the relative amount of residual aromatic compounds in petroleum waxes is the intensity of the blue fluorescence, which occurs when the material is exposed to UV radiation [18]. It was estimated both visually and by comparing the diffuse reflectance spectra corresponding to the exposure of the samples to the radiation of wavelength interval 360–700 nm and 400–700 nm (Section 2.2.4). 

#### 2.2.2. Physical Properties

The melting and cooling calorimetric curves were measured using the DSC 3+ instrument (Mettler Toledo, Greifensee, Switzerland). Samples of 7 mg were closed in aluminium crucibles and measured at atmospheric pressure under nitrogen atmosphere. The studied temperature interval was 1–99 °C, both heating and cooling rates were 2 °C∙min^−1^. For the temperature calibration, indium and zinc were used. The relevance of the calibration for lower-melting organic compounds was verified using nonadecane and tritriacontane. All samples were measured twice. The discussion was based on the results of the second run, thus eliminating the effect of the thermal history of the samples. 

Drop point measurement followed the standard method of ASTM D127–87 (1999). All samples were measured twice.

Viscosity of the molten samples at different temperatures was measured using rheometer AR-G2 (TA Instruments, Waters GmbH, Eschborn, Germany), operating in the coaxial cylinder geometry mode. The measurement was carried out at the temperature interval 65–91 °C, the shear rate was modulated within the interval 10^−4^–10^0^ s^−1^. Both heating and cooling curves were measured. 

Thermal volume expansion was measured by the hydrostatic weighing method. The samples were prepared in the form of spheres (30.0 ± 0.4 cm^3^ at 23 °C). After tempering in a degassed water bath, they were fitted to rigid suspension and weighed using laboratory balance. The weight of the suspension was sufficient to keep the wax samples submerged in water. The temperature interval studied was 5–35 °C, the heating rate was approximately 1 °C∙h^−1^. Two samples were prepared and measured for each material. 

Hardness Shore D was measured following the standard method of ISO 868:2003, using Shore D durometer HH-317 (Mitutoyo Akashi, Mitutoyo Europe GmbH, Neuss, Germany). For each sample, five measurements were carried out. 

The three-point flexural strength was measured using the Instron 3365 (Instron, Norwood, United States) testing machine equipped with the three-point flexural fixture. The procedure was based on the standard method of ISO 178:2001. Ten specimens were prepared from all the sample materials. The dimensions of the samples were 10 mm × 10 mm × 80 mm, the support span of the fixture 60 mm and the strain rate 1 mm∙min^−1^. 

To evaluate the resistance of the surface to the incrustation of impurities, special samples were prepared. One-half of the microscopic slides (thickness 1 mm) were coated by a layer of tested material (thickness 0.1 mm), whilst the second half were coated by “calibration material”, i.e., recent beeswax. A fine pigment (lamp black, 18729, H. Schmincke & Co.-GmbH & Co.KG, Erkrath, Germany) was then applied to the whole area of the coated slides using a soft brush, until the layer of “calibration material” reached the specified degree of soiling. The degree of contamination was evaluated both visually and by comparing the Status A blue diffuse reflection density, which was determined following the standard method of ISO 18916:2007 using X-Rite 811 densitometer (X-Rite, X-Rite Europe GmbH, Regensdorf, Switzerland).

Prior to all mechanical properties tests, samples were annealed for at least one month at 18 °C, and at the same temperature the measurement itself was carried out. This temperature corresponds to the typical conditions maintained in archival repositories in the Czech Republic.

#### 2.2.3. Working Properties

The processing properties of the wax mixtures were subjectively assessed by practical conservators of the National Archives in Prague, who specialize themselves in wax seals conservation. This assessment was comprised of the evaluation of plasticity by kneading the material with fingers, the applicability of the melt to the surface of the damaged seal model using electrically heated tools, and the casting properties and the mechanical shaping of the solidified material using a scalpel. 

#### 2.2.4. Stability

The stability of the materials was evaluated on the basis of results of two types of light ageing tests. For each ageing protocol, 1 mm thick platelets were prepared as samples. The exposure to UV-vis radiation was carried out employing the Q-Sun Xe-1 BC chamber equipped with xenon arc lamp Q-Lab X-1800, following the standard method of ISO 5630-7 (irradiance 0.45 W∙m^−2^ at 420 nm, radiant exposure 388 kJ∙m^2^, temperature 26 °C). The parallel ageing with the UV component of the radiation excluded was carried out in the same chamber, which was equipped with a UV blocking filter Window—B/SL (illuminance 40 klx, luminous exposure 4.8 Mlx∙h, temperature 26 °C) [23]. 

The chemical composition of the samples after the ageing was evaluated by the GC-MS method, the determination of the total acid number and by the evaluation of their fluorescence when exposed to the UV radiation (Section 2.2.1).

Optical properties (L*, a*, b* parameters of the CIELAB colour space, diffuse reflectance spectra) were measured using a spectrophotometer Konica-Minolta 2600 d (illuminant D65, observer 10°, aperture 3 mm, the specular component of reflected light excluded). The emission of blue visible light, which occurred as a consequence of the exposure of the samples to UV radiation was evaluated both visually and also qualitatively by comparing the diffuse reflectance spectra, which were acquired under different conditions (wavelength intervals of the illuminant 360–700 nm and 400–700 nm respectively). All of the measurements were carried out twelve times. 

Special samples were prepared for the evaluation of the incidence of the “wax bloom”, the whitish efflorescence on the surface of the material. The samples of wax were cast on watch glass and after the solidification, half of the surface was impressed with fingers. In this way, typical surface defects found on seals were created, which subsequently acted as nucleation centers of crystallization of the efflorescence components [24]. Samples were stored at 18 and 4 °C and visually evaluated. 

## 3. Results and Discussion

### 3.1. Chemical Composition

**Figure 2:**
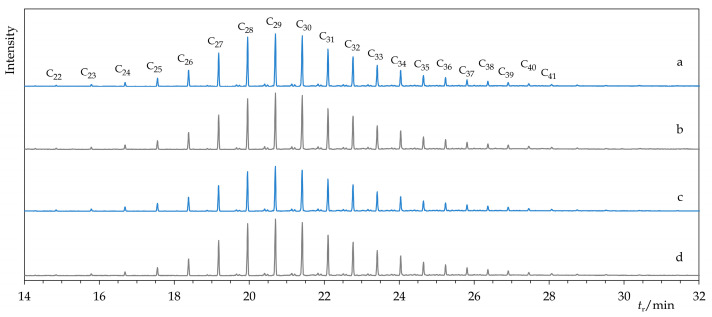
Comparison of chromatograms of Permulgin 3274 (**a**), hydrogenated Permulgin 3274 (**b**), UV aged Permulgin 3274 (**c**) and UV aged hydrogenated Permulgin 3274 (**d**). C*_x_*: *n*-alkanes with a chain length of *x* carbon atoms.

**Figure 3:**
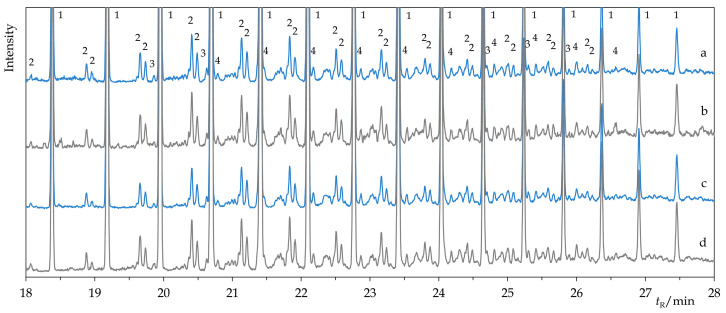
Comparison of the details of chromatograms of Permulgin 3274 (**a**), hydrogenated Permulgin 3274 (**b**), UV aged Permulgin 3274 (**c**) and UV aged hydrogenated Permulgin 3274 (**d**). 1: *n*-alkanes C_26_–C_40_, 2: isoalkanes, 3: alkylcyclopentanes, 4: alkylcyclohexanes.

The producer characterizes the composition of Permulgin 3274 as a mixture of paraffinic and isoparaffinic hydrocarbons, with chain length ranging from 20 to 70 carbon atoms [25]. In compliance with this statement, via the GC-MS analysis, it was possible to identify the typical series of *n*-alkanes (C_21_–C_45_, peak at C_29_) and isoalkanes as main constituents of the material. Because of their high boiling point, and probably also relatively low concentration, molecules featuring longer hydrocarbon chains (C_46_–C_70_) could not be detected using the current method. In addition, minor proportions of alkylcyclopentanes and alkylcyclohexanes were detected. From the point of view of the relative proportions and chain length distributions of these components, the material represents a transition between paraffins and microcrystalline waxes (Figure 2 and Figure 3).

One reason for the selection of Permulgin 3274 for this study was its expected high degree of purity. By the GC-MS method and the determination of total acid number, no impurities, admixtures or acid functional groups were detected. Nonetheless, the slight blue fluorescence, observable when the material was exposed to the UV radiation, revealed the presence of traces of residual aromatic compounds (Section 3.4.1, [18]). It was proven that their content can be efficiently reduced, and the fluorescence quenched by the hydrogenation.

The chemical composition of Permulgin 3274 and its hydrogenated derivative naturally differ from that of recent and historical beeswax, which are ester-type waxes [1]. Depending on the point of view, this difference can be regarded either as a drawback or an advantage. On the one hand, material authenticity is somewhat compromised, on the other hand, this very difference enables overcoming some limitations inherent to the traditional beeswax-based conservation mixtures. 

### 3.2. Physical Properties

#### 3.2.1. The Peak Melting Temperature

**Figure 4:**
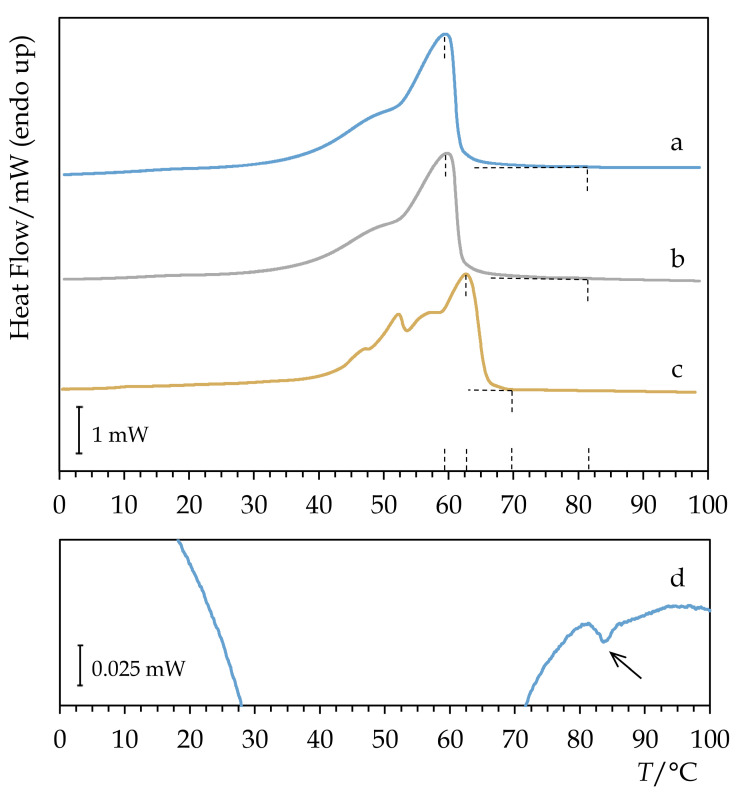
Comparison of DSC heating curves of (**a**) Permulgin 3274, (**b**) hydrogenated Permulgin 3274 and (**c**) recent beeswax. (**d**): Detail of the cooling curve of Permulgin 3274.

The calorimetric heating curve of Permulgin 3274 somewhat resembles that of recent and historical beeswax [1]. It detaches the baseline at c. 25 °C as a consequence of phase transitions of the lower-molecular components of the material, and, shortly after reaching the peak temperature of 60 °C, the material melts as a whole (Figure 4, Table 1). However, a small proportion of long chain molecules seem to retain a high degree of ordering up to c. 82 °C. Their solidification from the melt can be clearly observed on the cooling curve (Figure 4). The consequences of this property will be discussed in detail below.

#### 3.2.2. Drop Point

The drop point measurement further emphasized the difference between the melt properties of Permulgin 3274 and beeswax. In the case of beeswax, the drop point value exceeds the DSC melting peak temperature by approximately 2 °C, which is the expected result; in the case of Permulgin 3274, the difference reached 22 °C (Table 1). The presence of the long chain molecules in the Permulgin 3274 blend apparently increases the viscosity of the melt significantly.

#### 3.2.3. Viscosity of Molten Samples at Different Temperatures

**Figure 5:**
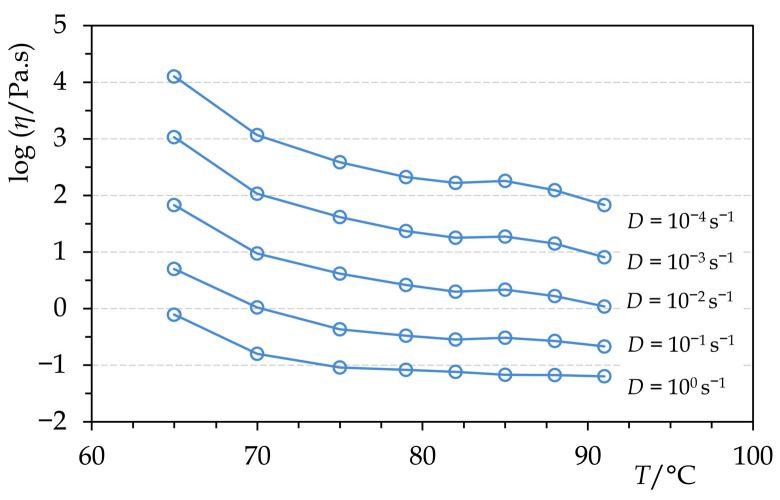
Temperature dependence of dynamic viscosity of molten Permulgin 3274 (cooling curves).

**Figure 6:**
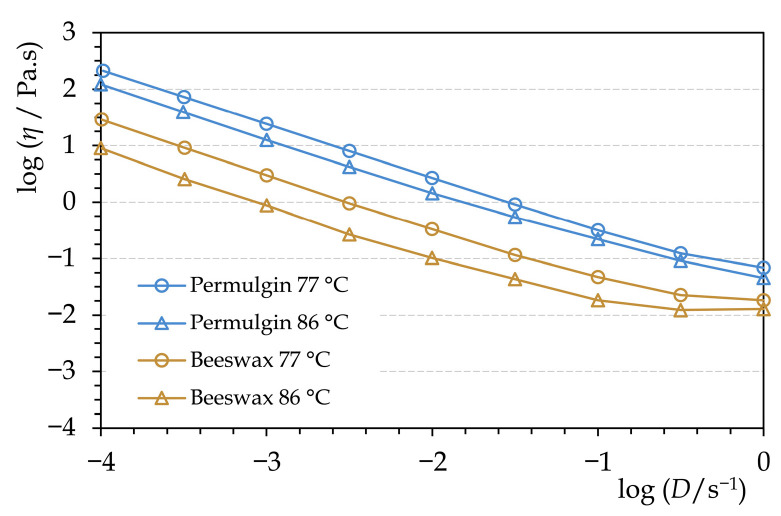
Comparison of rheograms of beeswax and Permulgin 3274 for two temperatures, below and above the drop point of Permulgin 3274.

The viscosity of molten Permulgin 3274 is generally negatively correlated with the increasing temperature. The exception is the temperature interval 82–85 °C, at which the viscosity is either nearly constant or even slightly increases (Figure 5). This could be related to the gradual increase in the hydrodynamic radius of the remaining semi-crystalline domains preceding their complete melting. At any rate, within the temperature interval of practical relevance, the viscosity of Permulgin 3274 melts is always substantially higher when compared to beeswax (Figure 6). This has several practical consequences. On the one hand, it reduces the sedimentation of pigments, which are routinely added to the melts for the colour adjustment; on the other hand, an increased temperature is required for ensuring adequate flow during casting processes (Section 3.3).

#### 3.2.4. Thermal Volume Expansion

**Figure 7:**
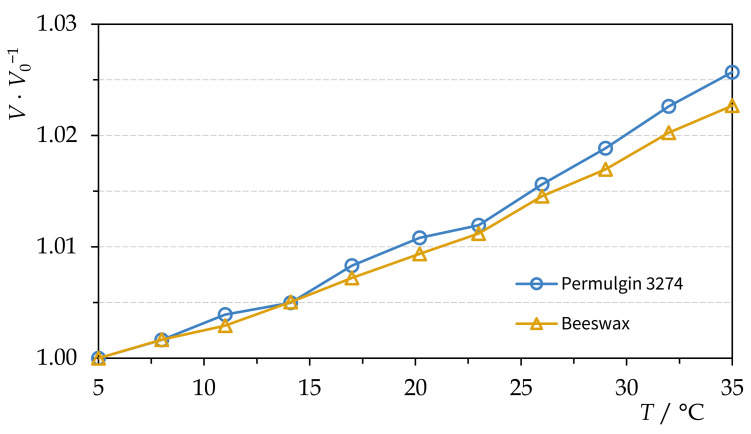
Thermal volume expansion of beeswax and Permulgin 3274.

It was found that, within the temperature interval 5–35 °C, the thermal volume expansion of beeswax and Permulgin 3274 did not differ significantly (Figure 7). Therefore, no stresses between the original and the added material should be expected, even under improbable fluctuating temperature conditions.

#### 3.2.5. Mechanical Properties

**Figure 8:**
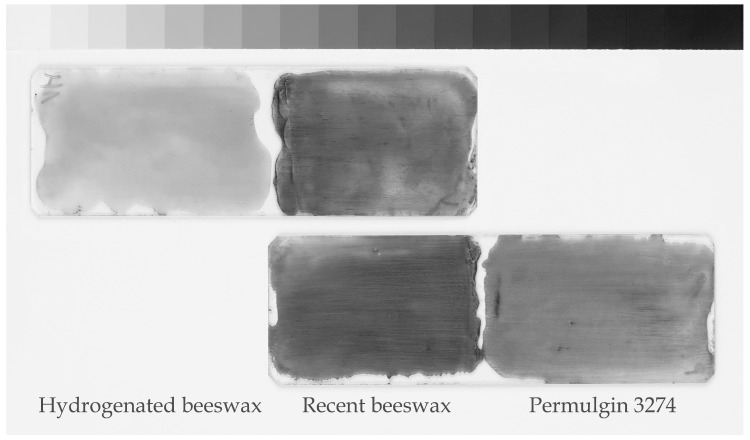
Incrustation of impurities into the surface of wax samples.

When accepting hydrogenated beeswax as a model material for naturally aged beeswax, it can be estimated that historical beeswax can be up to three times harder when compared to recent beeswax. The hardness Shore D of Permulgin 3274 then lies between the two extreme values (Table 2), reaching 149% and 52% of the hardness of recent and hydrogenated beeswax, respectively. This is actually favorable, making it less prone to deformation, abrasion and incrustation of impurities into the surface when compared to recent beeswax. The resistance to soiling increases in the order of recent beeswax, Permulgin 3274 and hydrogenated beeswax (Table 3, Figure 8). At the same time, the sufficient difference in hardness, when compared to the historical material, is retained. This is especially important when shaping the wax infills with mechanical working.

The qualitatively congruent results were obtained when measuring the three-point flexural strength and modulus. The flexural strength of Permulgin 3274 reaches c. 108% of the values of recent beeswax and 49% of the flexural strength of “historical”, i.e., hydrogenated, beeswax. By hydrogenation, the flexural strength of Permulgin increases by c. 30%. Permulgin 3274 is substantially more brittle than recent beeswax, but still less when compared to hydrogenated beeswax (Table 2). 

## 3.3. Working Properties 

This section summarizes the practical experience gained by the conservators of the National Archives in Prague during the work with Permulgin 3274. 

### 3.3.1. Mechanical Processing

As far as malleability is concerned, Permulgin 3274 resembles beeswax, although its hardness makes kneading with the fingers a bit more laborious. The mutual adhesion of layers of kneaded material is significantly better than in the case of paraffins, whilst it does not exhibit excessive stickiness of microcrystalline waxes of the comparable melting temperature. 

The applicability of Permulgin 3274 to the surface of the damaged seal, using electrically heated tools, does not significantly differ from that of traditional beeswax-based mixtures. The increased viscosity of the melt makes it even easier to control its flow.

During casting processes, it is advisable to heat the melt up over 82 °C to lower its viscosity and to improve its homogeneity and flow properties. During the solidification from the melt, one should expect a similar volume contraction as in the case of beeswax.

The infills shaping made of Permulgin 3274 with mechanical working is comfortable. Chips and shavings of wax do not tend to stick to the working tool.

### 3.3.2. Colour Adjustment

**Figure 9:**
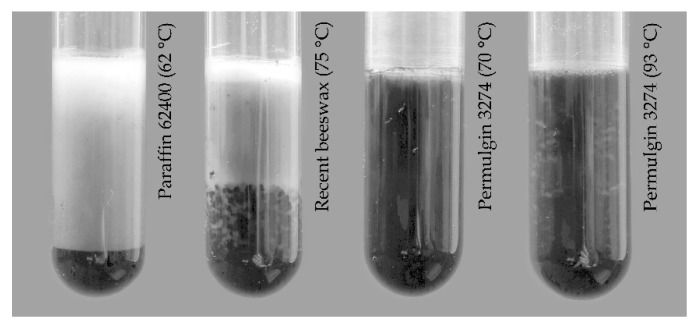
Comparison of sedimentation extent of pigments in molten waxes at different temperatures after 20 min. Used temperatures are 10 °C higher than melting temperature/drop point.

The colour of historical sealing wax can vary within wider limits than natural grades of waxes provide. Therefore, the colour of materials used for the conservation is often adjusted using inorganic pigments. In the case of Permulgin 3274, it was found that the content of pigments generally should not exceed c. 1 % *w/w*, to avoid unnatural opacity of the material. Only in the cases when imitating coloured, opaque sealing wax, can the greater amount of pigments (e.g., 20% *w*/*w*) be recommended. The sedimentation of pigments in molten Permulgin 3274 is slower than in the case of lower viscosity waxes, including beeswax, as already mentioned in Section 3.2.3 (Figure 9).

### 3.3.3. Fluorescence

**Figure 10:**
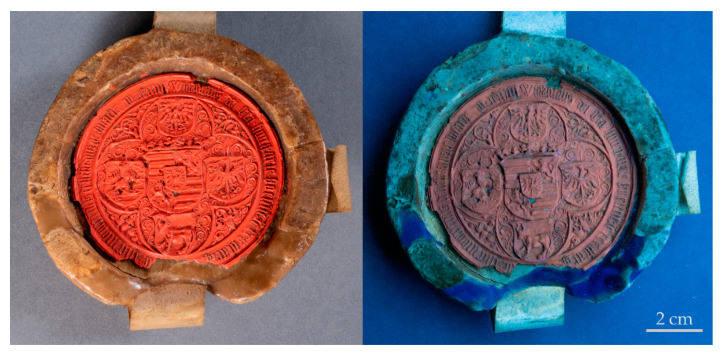
Seal of the charter CG-L 494 partially reconstructed using coloured Permulgin 3274. (**Left**): photograph acquired under visible light, (**Right**): photograph acquired under UV light (lamp: OSRAM L18 W/73). Photographs courtesy of the National archives in Prague.

Any material used in the course of the conservation treatment should be ideally distinguishable and removable from the original, if required. Permulgin 3274 exhibits a blue fluorescence when exposed to UV radiation. Since the fluorescence is easily visually observable, it could be exploited by the conservators, both for the identification and the control of the completeness of the possible removal of the infill (Figure 10). Nonetheless, this favourable property is lost after the prolonged exposure to UV radiation or during the hydrogenation process. 

The possible removability of the infill can be facilitated by the use of a suitable separation layer between Permulgin 3274 and the original, e.g., a solution of poly(2-ethyl-2-oxazoline) in ethanol. 

## 3.4. Stability

The stability of compared materials was estimated on the basis of the changes in the chemical composition and optical properties, which took place during ageing experiments. These experiments involved the exposure to UV-vis and visible radiation and long-term storage of the samples under repository conditions.

### 3.4.1. Chemical Composition

**Figure 11:**
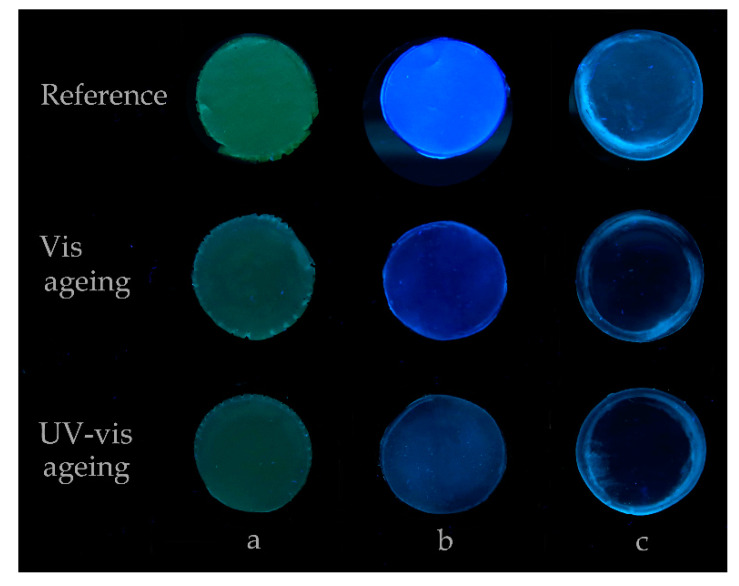
Comparison of the fluorescence of the samples before and after the exposure to UV-vis radiation for 240 h. (**a**): recent beeswax, (**b**): Permulgin 3274, (**c**): hydrogenated Permulgin 3274. Photograph acquired under UV light (lamp: OSRAM L18 W/73).

As a consequence of the exposure of Permulgin 3274 to the UV-vis radiation for 240 h, signs of oxidation were detected. These comprised the measurable increase in the total acid number, i.e., the increase in content of acid functional groups (Table 4), and the decrease in the fluorescence of the material exposed to UV radiation, i.e., the change in the aromatic compounds’ content (Figure 11, Section 3.4.2). 

However, these changes were not detectable by the GC-MS method and can be considered negligible in terms of the intended use of the material (Figure 2 and Figure 3). The chemical stability of the hydrogenated derivative of Permulgin 3274 was still superior, judging by the lower total acid number of the aged samples (Table 4). 

### 3.4.2. Optical Properties

**Figure 12:**
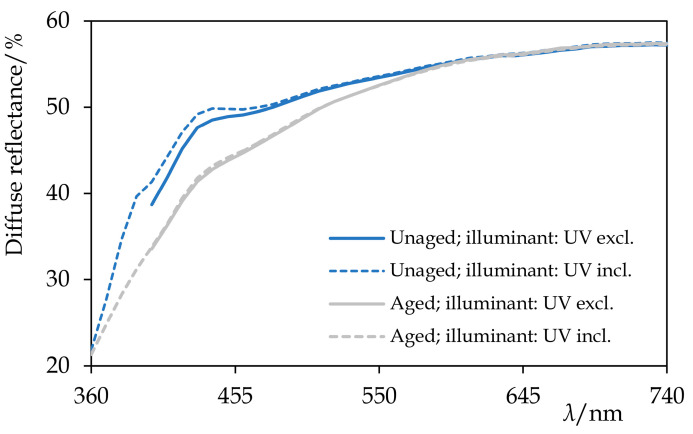
The diffuse reflectance spectra of Permulgin 3274 before and after the artificial ageing by the exposure to UV-vis radiation for 240 h. Information about the illuminant used during the acquisition of the spectra is given. Note the yellowing and the decrease in the fluorescence of the aged sample.

The colour changes, which took place during the exposure to the UV-vis radiation, reflected the changes in the chemical composition. After the first 120 h of ageing, Permulgin 3274 showed a slight tendency towards yellowing and darkening Δ*E*_ab_* = 3.0. The rate of the discolouration decreased with time, however, and during the subsequent 120 h of ageing, it was more than compensated by a slight bleaching (Table 5, Figure 12). Besides, after 240 h of the UV-vis ageing, samples of Permulgin 3274 did not exhibit further intensive fluorescence when subsequently exposed to UV radiation. This change could be observed both visually (Figure 11) and could also be demonstrated by the comparison of the differences in the diffuse reflectance spectra, measured prior to and after the ageing experiment, under different conditions (wavelength intervals of the illuminant 360–700 nm and 400–700 nm, respectively; Figure 12).

**Figure 13:**
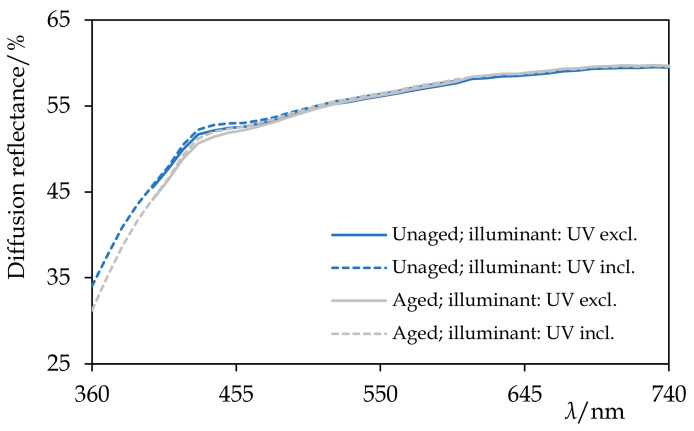
The diffuse reflectance spectra of hydrogenated Permulgin 3274 before and after the artificial ageing by the exposure to UV-vis radiation for 240 h. Information about the illuminant used during the acquisition of the spectra is given. Note the absence of the fluorescence and the excellent stability of the sample.

The superior stability of hydrogenated Permulgin 3274 was confirmed both by the evaluation of its diffuse reflectance spectra and the lower total colour difference (Table 5, Figure 13). Furthermore, the possible addition of pigments to Permulgin 3274 would generally shield the wax matrix and improve its photooxidative stability [26,27]. The complementary ageing procedure with UV components of the radiation blocked did not lead to substantial changes, confirming the decisive importance of high energy UV radiation for the oxidation process (Table 5). 

**Figure 14:**
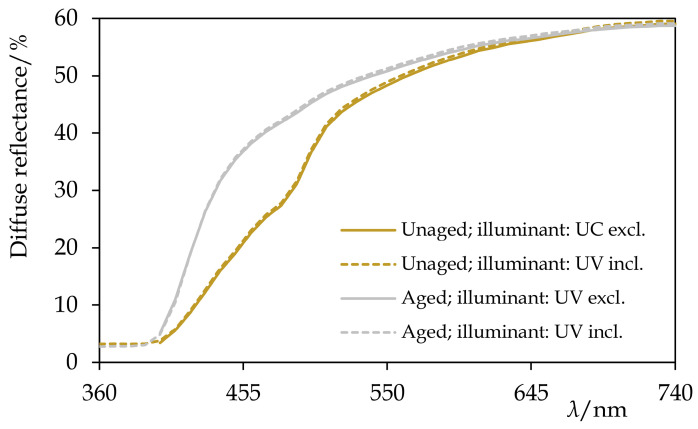
The reference diffuse reflectance spectra of recent beeswax before and after the artificial ageing by the exposure to UV-vis radiation for 240 h. Information about the illuminant used during the acquisition of the spectra is given. Note the pronounced bleaching of the sample. Based on the data taken from [2].

The reference beeswax sample, however, showed the opposite trend. The total colour difference after 120 h of ageing was much more significant (Δ*E*_ab_* = 14.6) and was primarily related to bleaching and the decrease in yellowness (Table 5, Figure 14).

From a practical point of view, the stability of mixtures based on Permulgin 3274 can be probably considered as an advantage, because it provides the conservator greater control over the properties of the material. As the long-term exposure of parchment charters and seals should be out of the question, the risk of deterioration of colour match between the conservation and the original material should be acceptable.

### 3.4.3. “Wax Bloom”

As expected, the samples made of beeswax or hydrogenated beeswax were liable to develop “wax bloom”. This term denotes a more or less uniform whitish crystalline layer that develops on the surface of beeswax objects under specific conditions. The “wax bloom” is generally considered undesirable, because it compomises the aesthetic and sometimes even informational value of the affected objects. The chemical composition of the efflorescence on pure beeswax has been recently studied in detail. It was found that it consists primarily of a mixture of linear monounsaturated hydrocarbons and a small proportion of saturated hydrocarbons. These are natural constituents of beeswax [3].

**Figure 15:**
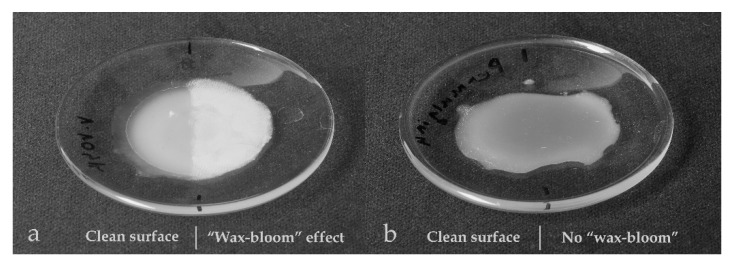
Comparison of the samples in terms of their resistance to the “wax bloom” phenomenon at 18 °C. (**a**): beeswax sample, (**b**): pigmented Permulgin 3274 sample. The right half of each sample was impressed with fingers. In the case of beeswax, this half is covered by the whitish efflorescence.

The whitish efflorescence appeared on the newly created surfaces of the samples within several months, starting from the surface defects, e.g., fingerprints. Permulgin 3274 samples, though, remained unaltered under the same repository conditions (18 °C, 50% RH) for 3 years, which can be regarded as one of the important advantages of its possible use (Figure 15).

## 4. Conclusions

The characterization of Permulgin 3274 and its hydrogenated derivative proved that, from a technological point of view, these materials could be regarded as a welcome alternative to traditional wax mixtures used for the conservation of historical beeswax seals. The main advantages and limitations of their use can be summarized as follows:

Advantages:

1. Absence of the “wax bloom” phenomenon
2. Distinguishable from the original on the basis of its fluorescence, which occurs when exposed to the UV radiation (not in the case of the hydrogenated derivative)
3. Relatively high viscosity of its melt reduces the sedimentation rate of pigments added to the mixture
4. Very good stability, even when exposed to light (especially the hydrogenated derivative)
5. Adequate hardness and bending strength.

Limitations:

1. Necessity of colour adjustment
2. Somewhat higher temperatures are required during the casting processes
3. Not so naturally available when compared to beeswax
4. Inauthentic material.

Probably the most severe objections could be related to its “historical incompatibility”. Of course, the decision to what extent, and at what cost, to insist on the ideal of material authenticity is always subjective and dependent on the context. In one area, however, its use can be clearly and unquestionably recommended. This is in the production of facsimiles, where its lightfastness and the absence of the “wax bloom” phenomenon, especially, are of great advantage.

## Figures and Tables

**Table 1:** DSC melting temperature and drop point of Permulgin 3274, beeswax and their hydrogenated derivatives.

Material	*T*_m_ (Peak)/°C	Drop Point/°C
Permulgin 3274	60	82
Permulgin 3274 (hydrogenated)	60	83
Beeswax	63	66
Beeswax (hydrogenated)	65	67

**Table 2:** Comparison of mechanical properties of Permulgin 3274, beeswax and their hydrogenated derivatives. The data are expressed as mean ± standard deviation. The reference data for beeswax and hydrogenated beeswax were taken from [2].

Material	Hardness Shore D/15 s	Flexural Strength/ MPa	Flexural Modulus/ MPa	Flexural Strain at Break/ %
Permulgin 3274	10.6 ± 0.9	2.7 ± 1.2	272 ± 38	2.8 ± 0.2
Permulgin 3274 (hydrogenated)	10.2 ± 0.8	3.5 ± 0.2	310 ± 47	3.7 ± 0.5
Beeswax	7.1 ± 0.4	2.5 ± 0.1	255 ± 31	12.8 ± 2.2
Beeswax (hydrogenated)	20.4 ± 0.8	5.5 ± 0.8	757 ± 53	1.2 ± 0.2

**Table 3:** Difference of the Status A blue diffuse reflection density (*D*_R_) of the wax films measured before and after the incrustation of the pigment into the surface. The data are expressed as mean ± standard deviation.

Material	Δ*D*_R_
Permulgin 3274	0.95 ± 0.08
Recent beeswax	1.45 ± 0.09
Hydrogenated beeswax	0.55 ± 0.09

**Table 4:** Total acid number before and after the UV-vis ageing experiment. The data are expressed as mean ± standard deviation.

Material	Unaged	UV-Vis Aged
Permulgin 3274	0.0 ± 0.00	0.50 ± 0.10
Permulgin 3274 (hydrogenated)	0.01 ± 0.01	0.33 ± 0.01

**Table 5:** The parameters L*, a*, b* and the total colour difference of samples after the exposure to UV-vis and visible radiation for 120 and 240 h respectively. The data were expressed as mean ± standard deviation. Δ*E*_ab_* values are related to the unexposed control samples both in the case of 120 h and 240 h exposure.

Parameter	Ageing Type	Exposure	Permulgin 3274	Permulgin 3274 (Hydrogenated)	Recent Beeswax
L*	Reference	0 h	81.30 ± 0.17	82.33 ±0.19	72.13 ± 0.16
	UV-vis aged	120 h	79.74 ±0.19	82.52 ± 0.27	72.65 ± 0.07
		240 h	80.03 ± 0.07	82.68 ± 0.35	73.38 ± 0.04
	Vis aged	120 h	81.95 ± 0.22	83.09 ± 0.08	73.32 ± 0.25
		240 h	81.79 ± 0.25	83.01 ± 0.17	73.85 ± 0.11
a*	Reference	0 h	0.58 ± 0.03	0.51 ± 0.04	−1.87 ± 0.03
	UV-vis aged	120 h	0.45 ± 0.01	0.45 ±0.03	−0.36 ± 0.05
		240 h	0.44 ± 0.01	0.46 ± 0.01	−0.46 ± 0.02
	Vis aged	120 h	0.55 ± 0.01	0.47 ± 0.03	−0.88 ± 0.07
		240 h	0.54 ± 0.01	0.49 ± 0.03	−0.89 ± 0.04
b*	Reference	0 h	4.26 ± 0.07	3.56 ± 0.09	49.25 ± 0.13
	UV-vis aged	120 h	6.84 ± 0.05	4.12 ± 0.06	34.75 ± 0.33
		240 h	6.84 ± 0.04	4.15 ± 0.04	31.55 ± 0.06
	Vis aged	120 h	4.44 ± 0.03	3.53 ± 0.06	34.20 ± 0.57
		240 h	4.61 ± 0.03	3.56 ± 0.12	30.24 ± 0.20
Δ*E*_ab_*	UV-vis aged	120 h	3.0 ± 0.1	0.6 ± 0.1	14.6 ± 0.4
		240 h	2.9 ± 0.1	0.7 ± 0.2	17.8 ± 0.1
	Vis aged	120 h	0.7 ± 0.3	0.5 ± 0.3	15.1 ± 0.6
		240 h	0.6 ± 0.2	0.4 ± 0.3	19.1 ± 0.2

L*—the lightness coordinate of the CIELAB color space (black: 0, white: 100). a*—the a coordinate of the CIELAB color space (green: −a, red: +a). b*—the b coordinate of the CIELAB color space (blue: −b, yelow: +b). Δ*E*_ab_*—the total color difference (CIE76).

## Data Availability

Not applicable.
